# Multiple Fitness Improvements Found after 6-Months of High Intensity Functional Training

**DOI:** 10.3390/sports7090203

**Published:** 2019-09-02

**Authors:** Sarah J. Cosgrove, Derek A. Crawford, Katie M. Heinrich

**Affiliations:** 1 Department of Kinesiology, Kansas State University, Manhattan, KS 66506, USA; 2 School of Nutrition, Kinesiology, and Psychological Sciences, University of Central Missouri, Warrensburg, MO 64093, USA

**Keywords:** CrossFit, exercise, strength, power, endurance

## Abstract

While short-term high intensity functional training (HIFT) effects have been established, fitness improvements from program participation exceeding 16 weeks are unknown. This study examined the effectiveness of participation in HIFT through CrossFit. During 2013–2014, fitness performance testing was incorporated into an ongoing university CrossFit program. Participants included 45 adults (23 women, 22 men) with 0–27 months of HIFT experience (grouped into 0–6 months and 7+ months). Participants completed three separate days of assessments across 10 fitness domains before and after participating in the program for six months. For each sex, 2 (Time) × 2 (Group) RANOVA were used for each fitness test. For women, significant Time effects were found for four fitness domains (i.e., flexibility, power, muscular endurance, and strength), and a Group × Time interaction for cardiorespiratory endurance, with the 0–6-month group improving more. For men, significant Time effects were found for flexibility, muscular endurance, and strength. These data provide evidence for multiple fitness improvements after six months of CrossFit participation with greater 1.5 mile run time improvement among women with less experience.

## 1. Introduction

Physical inactivity and lack of physical fitness persist as major public health concerns with many United States (US) adults at risk for chronic disease and early mortality [1,2,3]. Exercise participation is as a primary preventive strategy for combating these negative health trajectories [4]. Further, evidence suggests combined exercise programs (i.e., those that include both aerobic and resistance training strategies) that emphasize high intensities may offer the best opportunity for maximizing physical health and function to offset this public health burden [5,6,7,8]. However, participation in combined exercise programs remains challenging as many within the general population report significant barriers in meeting both aerobic and resistance training recommendations [9,10] and almost half of participants drop out within the first six months [11]. Two of the primary barriers to participation are a lack of time or enjoyment of specific exercise modalities or intensities (e.g., resistance training or high-intensity programs) [12,13,14]. Identifying exercise interventions or programs that combine exercise modalities within an enjoyable and time-efficient framework should warrant considerable interest from those looking to improve public health. 

High intensity functional training (HIFT) is an exercise program which emphasizes foundational, or “every day,” movements (e.g., lifting, pulling, and locomotor movements) while temporally combining both aerobic and resistance training elements performed at high intensities [15]. HIFT programs continue to grow as the focus on improving “general physical preparedness” in a time efficient manner appeals to a wide audience, whether it be athletes or the general population [16,17]. Rather than focusing on one specific aspect of fitness (e.g., cardiovascular endurance), HIFT’s emphasis on general physical preparedness prioritizes broad adaptation across multiple physical domains rather than specificity within any one single domain [18]. Ten physical skills contributing to one’s general physical preparedness are emphasized including cardiovascular endurance, stamina, muscular strength and power, flexibility, speed, coordination, agility, balance, and accuracy [18]. More importantly, these key aspects of physical fitness are positively correlated with health-related quality of life across age groups, genders, and multiple chronic health conditions [19]. 

Much of the research around HIFT interventions, thus far, centers on its effects on many of these general physical skills [20,21,22,23,24]. While the existing literature on HIFT highlights its utility in these endeavors, a recent meta-analysis suggests that few, if any, high-quality and low bias studies exist within this field [25]. Further, these relatively short-term (i.e., 3–16 weeks) interventions, lack of comprehensive outcome assessments (i.e., most general physical skills), and lack of studies on real-world HIFT programs (e.g., CrossFit) employed in these studies leaves many unanswered questions with respect to chronic HIFT participation. Recently, Box et al. [26] found the length of HIFT experience was positively associated with greater motivation for relatedness and enjoyment, and negatively associated with weight management. Age may also influence motives for HIFT participation, with older adults (≥50 years) reporting higher motives for avoiding ill health and weight management and younger adults (18–24 years) reporting higher social, affiliation, competition, stress management, and challenge motives [27]. It is plausible that these differences in motivation may be the result of experiencing changes in fitness from HIFT participation leading to potential questions such as identifying if HIFT outcomes differ between age groups or experience levels. Addressing these questions will allow for a more complete understanding of HIFT under the broad scope of exercise programs and its potential benefit within a public health context. 

The current study was undertaken to examine the effectiveness of HIFT via an existing community CrossFit program for adults. Similar to other studies, periodic fitness measures were included as part of the ongoing program to monitor quality, provide objective feedback to program participants regarding fitness levels, and provide evaluation data for program administrators [28]. This paper presents data on differences in multiple fitness domains (i.e., balance, coordination, accuracy, agility, flexibility, power, stamina/muscular endurance, strength, and cardiovascular/respiratory endurance) over six months of participation in CrossFit. Similar to previous CrossFit research we conducted sex specific analyses [21], as well as compared differences by length of previous CrossFit experience. We hypothesized that there would be improvements across all general physical fitness skills assessed and that these changes would be similar between previous CrossFit experience groups.

## 2. Materials and Methods

### 2.1. Participants

For study inclusion, participants had to be age 18 or older, new or current members of K-State CrossFit, and plan to continue their CrossFit participation. No exclusion criteria were specified. K-State CrossFit members were recruited on a volunteer basis for the program evaluation. Participants provided written, informed consent. In accordance with the Declaration of Helsinki, all study protocol and procedures were approved by the university’s institutional review board (#6046). 

Participants included in this paper attended one of four initial assessment periods (i.e., February, April, August, or October 2013), completed follow-up assessments 6 months later, and provided duration of CrossFit experience at baseline. This included a total of 45 participants (51.1% women, mean age = 31.8 ± 13.2 years, mean weight = 72.5 ± 13.6 kg, mean height = 1.73 ± 0.10 m). The majority were non-Hispanic Caucasians (87.5%). Participants’ experience with HIFT ranged from 0–27 months, with an average of 9.8 (SD = 8.4) months. Participants reported attending HIFT classes 4.0 (SD = 1.1) days per week, with a range of 2–6 days per week. Participant demographic characteristics by experience are displayed for each sex in Table 1.

### 2.2. Exercise Program

CrossFit is a group-based exercise program involving coach-supervised 60-min classes. The general class format includes a warm-up (10–15 min), skill work and technique practice (10–20 min), a workout (5–30 min), and cool-down stretching (5 min) [29]. Workouts are designed utilizing the CrossFit template, which uses a constantly varied combination of aerobic (e.g., running), body weight (e.g., pull-ups), and weightlifting (e.g., kettlebell swings) exercises that are completed for time, repetitions, distance, or weight lifted [18]. The goal of the program is to develop general physical preparedness across multiple fitness domains rather than emphasizing one type of fitness (e.g., aerobic endurance) over another [17]. 

As noted in Table 1, participants reported completing workouts two to six days per week with each participant choosing the number of days per week that they engaged in the workouts. Actual attendance was not tracked. The constantly varied workouts were performed at a relatively high-intensity, which was self-selected. All classes occurred either inside a 4000 ft^2^ temperature-controlled gymnasium, a 200-m indoor rubberized track, or outdoors on a 400-m rubberized track with an artificial turf infield. Coaches had either a CrossFit Level 1 or Level 2 certificate.

### 2.3. Measures

In workout clothing and without shoes, participant height was measured with a Seca Stadiometer (Seca North America, Chico, CA, USA) to the nearest 0.5 cm. Weight was measured with a calibrated scale to the nearest 0.1 kg. These measurements were used to calculate body mass index as weight in kg divided by height in m^2^.

All fitness tests were supervised by CrossFit-L1 trainers and research staff members who were first aid and CPR/AED certified. For each fitness test session, participants completed a standardized warm-up which consisted of 5 min of aerobic activity (e.g., light running, rowing, jumping rope) followed by a series of full-body, dynamic stretches [30]. 

Physical fitness tests were used to measure changes in participant’s performance in concert with the 10 general physical skills in nine fitness domains (see Table 2). Tests were separated into three successive days. In the event a participant missed one of the testing days, a make-up testing session was scheduled. 

#### 2.3.1. Session 1 Fitness Tests

Coordination and balance were assessed using the agility hop test [32]. Participants were asked to hop forward and laterally (zigzag pattern) on one foot to a series of 6 tape marks holding a stable and balanced landing position for five seconds after each hop. Hands were to stay at the participant’s side and their contralateral leg lifted. Errors were recorded for the participant for every adjustment made between jumps (e.g., moved the test foot or did not “stick” the landing, did not remain on each spot for five seconds). Participants were allowed to repeat the test twice (if desired), but were required to use the same foot for each test. 

Agility was assessed using the pro-agility test [33]. Participants were timed as they ran a 20-yard (18.3 m) shuttle sprint. The test was initiated with participant facing forward with hips squared in a two-point stance straddling the starting line. On the command “Go” the participant turned 90 degrees to their left and ran 5 yards, touched a line with their foot and turned 180 degrees, ran 10 yards to a second line, touched the line with their foot and turned 180 degrees again, and ran 5 yards back across the starting line. Participants were allowed to repeat the test twice (if desired), as long as there was at least 3 min of recovery between attempts. The best time to complete the shuttle run was recorded. The pro-agility test has shown high reliability (*r* = 0.91) for predicting forward agility [34].

Flexibility was assessed using a Novel Flex-Tester^®^ Trunk Flexibility Tester (Novel Products, Inc., Rockton, IL, USA). Participants sat with their legs straight out in front, toes pointed upward and shoes off with heels against the foot plate. Participants overlapped their hands and maintained contact with the box during the reach, pushing the marker as far as possible without bending their knees. The best of three attempts were recorded to the nearest half cm. The sit and reach test has shown high reliability (*r* = 0.96–0.98) and validity (*r* = 0.24–0.53, *p* < 0.05) for predicting hamstring flexibility across genders [35].

Lower body power was assessed using the standing long jump [34]. Participants were required to stand with feet parallel and squared to the starting line and then execute a max effort long jump with a countermovement arm swing prior to jumping. Participants were scored based on the distance between the starting line to the back of their nearest heel measured to the nearest ½ inch; values were converted to meters. Participants were given three attempts with adequate rest for recovery between each; the furthest attempt was used. The standing long jump has shown high reliability (*r* = 0.83–0.86) and validity (*r* = 0.24–0.53, *p* < 0.001) for predicting lower body power in youth [36].

Upper body power was assessed with the medicine ball put [37]. Participants were seated with their back and shoulders upright and against the back of an adjustable incline bench with feet flat on the ground. This position was standardized in all attempts to ensure greater stability and minimize movement of the trunk during the performance. Men used a 20-lb (9.1 kg) medicine ball while women used a 14-lb (6.4 kg) medicine ball. Participants were given 3–5 attempts to propel or “put” the ball as far as possible at a 45-degree angle from their chest, with their shoulders and back staying against the bench. The maximum distance measured to the nearest ½ inch as the distance between landing spot closest to participant and the front of the bench was recorded; values were converted to cm. The medicine ball put has shown excellent test–retest reliability for men and women (*r* = 0.86 and 0.79, *p* < 0.05) [37].

Muscular endurance was assessed with using three 60-s tests: push-ups [38], sit-ups [34], and bodyweight squats [39] with full recovery between each test. For the push-up test, participants began in a prone planked position, lowered themselves to the floor until their chest and thighs touched and then pushed themselves up with a straight body until achieving elbow extension. Participants were allowed to modify their push-ups by doing them from their knees, but were required to use this modification at the subsequent testing period. Of note, the only participants who chose the modification were women. For the push-up test, participants were instructed to maintain a neutral spine through the entire movement. The push-ups test has shown high reliability (*r* = 0.95 and 0.91) for predicting upper body muscular endurance in college-aged men and women [38]. Sit-ups were completed with the participants lying supine on the floor with their feet secured with dumbbells and legs at a 90-degree angle. Participants had to touch the ground behind their head with their hands or finger, while their shoulders remained in contact with the ground, and then rise up and touch the wall in front of them with their hands or fingers. Squats were performed as a bilateral movement and were defined as bending of the legs and hips until the participant’s hip crease reached a point below parallel to the knee, and then the participant fully extended both their hip and knee to stand. A 30-s, bilateral squat test has shown moderate test–retest, intraclass correlation coefficients (ICC = 0.79) in adults [39]. For all three 60-s tests, the total number of repetitions completed were recorded.

Upper body pulling endurance was assessed using the pull-up test [40]. Participants began the test with a shoulder-width full hang on the pull-up bar with palms facing away from them. A successful repetition was when participants pulled themselves from a full hang to chin over the bar and returned to the starting position without body swinging or kipping. If necessary, participants could modify the pull-up by using an elastic band; that modification was then used for each subsequent testing period. The sequence of movement was repeated to voluntary exhaustion. 

#### 2.3.2. Session 2 Muscular Strength Tests

Strength was assessed by finding a one repetition maximum (1RM) for three lifts (i.e., back squat, standing press, and deadlift) using a standardized protocol during Session 2 [34]. Participants completed the same percentage-based warm-up pattern for each lift; 10 repetitions of the given lift at 50% of their previous max weight (values were estimated at baseline), followed by 5 repetitions at 70% 1RM, 3 repetitions at 80% 1RM, and 2 repetitions at 90% 1RM. Following this repetition scheme for warmup, subjects were allowed up to 3 attempts to establish each of the identified 1RMs. Values were recorded in kilograms (kg). 

A successful back squat was classified by the participant’s hip crease passing below parallel to his or her knee, followed by full knee and hip extension at the top of the lift. The 1RM back squat has shown high test–retest reliability (*r* = 0.92–0.99) for predicting lower body muscular strength in recreationally active and resistance trained men [40]. A successful standing press was classified by the participant pressing the barbell to full extension of the elbow above his or her head, with no bending of the knee or hip during the movement. The 1RM shoulder press has shown high test–retest, intraclass correlation coefficients (ICC = 0.94–1.00) for both men and women [41]. A successful deadlift was classified by the participant lifting a barbell from the ground to full hip and leg extension, with the participant’s hands placed outside of his or her feet. 

#### 2.3.3. Session 3 Cardiovascular/Respiratory Speed and Endurance Tests

Cardiovascular/respiratory speed and endurance were assessed on a 200-m indoor rubberized track. Participants were first tested for speed by completing a 400-m sprint with time to completion recorded to the nearest 0.1 s. After 10–15 min of rest and recovery, participants ran 1.5 miles (2400 m). Participant times from the 1.5 mile run were used to estimate VO_2_max based on the Cooper [31] 1.5-mile run/walk test using the following equations for women and men, respectively: VO_2_max (mL·kg^−1^·min^−1^) = 88.020 − (0.1656 × body mass in kg) − (2.767 × time in minutes) and VO_2_max (mL·kg^−1^·min^−1^) = 91.736 − (0.1656 × body mass in kg) − (2.767 × time in minutes). 

### 2.4. Statistical Analysis

Data were entered into SPSS 25 (IBM SPSS, Armonk, NY, USA) for analysis. Descriptive statistics were run for demographic characteristics, including means, standard deviations, and percentages. Data distributions were examined for outliers. Statistical significance was set at *p* ≤ 0.05. All analyses were stratified by sex given well established findings of sex-based performance differences in fitness and because some tests were sex-specific. Months of HIFT experience (range = 0–27 months) were divided into two groups for 0–6 months and 7+ months based on research showing around 50% of exercisers drop out within the first 6 months [11]. We examined changes in fitness during the 6 months of CrossFit participation using a conservative Intention-to-Treat (ITT) model, where baseline values were carried forward [42], using a 2 × 2 (Group × Time) repeated-measures ANOVA (RANOVA). 

## 3. Results

### Fitness Test Results by Experience Group for Each Sex

Results for baseline and six-month fitness tests for women are shown in Table 3. A significant main effect of time was found for sit-and-reach (ƒ(1,21) = 13.8, *p* = 0.001, η^2^ = 0.40), standing long jump (ƒ(1,21) = 7.57, *p* = 0.012, η^2^ = 0.27), push-ups (ƒ(1,12) = 5.62, *p* = 0.035, η^2^ = 0.32), back squat (ƒ(1,21) = 24.93, *p* < 0.001, η^2^ = 0.67), press (ƒ(1,21) = 11.09, *p* = 0.003, η^2^ = 0.35), and deadlift (ƒ(1,21) = 8.12, *p* = 0.010, η^2^ = 0.28), with improvements from pre-test to post-test. No significant main effects for were found for Group. A Group × Time interaction was found for 1.5-mile run. The interaction reflected faster 1.5 mile run times for the less experienced group (0–6 months) as compared to the more experienced group (7+ months). 

Results for baseline and 6-month fitness tests for men are shown in Table 4. A significant main effect of time was found for sit-and-reach (ƒ(1,20) = 5.56, *p* = 0.029, η^2^ = 0.22), pull-ups (ƒ(1,15) = 6.60, *p* = 0.021, η^2^ = 0.31), back squat (ƒ(1,20) = 24.69, *p* < 0.001, η^2^ = 0.55), and deadlift (ƒ(1,20) = 16.16, *p* = 0.001, η^2^ = 0.45), with improvements from pre-test to post-test. No significant main effects for Group or Group × Time interactions were found.

## 4. Discussion

This study examined the effectiveness of an existing community CrossFit program for adults, comparing differences by length of CrossFit experience for each sex. We hypothesized that after six months women and men participants would achieve improvements in test scores in all fitness domains, regardless of length of experience at enrollment. The results of our program evaluation partially supported the hypothesis. From baseline to six months for women, a significant effect of Time was found for flexibility (sit-and-reach), power (long jump), muscular endurance (push-ups), and strength (back squat, press, and deadlift). Additionally, there was a significant Group × Time interaction, where women with less experience had significantly greater improvements in cardiovascular/respiratory endurance (1.5-mile run) than those with greater experience. For men, only a significant effect of Time was found for flexibility (sit-and-reach), muscular endurance (pull-ups), and strength (back squat and deadlift).

Improvements in flexibility were noted in the present study for both males and females on the sit-and-reach test. Similarly, Zagdursen et al. [22] showed significant increases in the sit-and-reach test for (*p* = 0.002; 35.4 ± 6.28 vs. 37.5 ± 5.16 cm) both males and females. Unfortunately, at the time of publication, only one other study has investigated the effects of HIFT on flexibility using the sit-and-reach test [43]. However, the age of this study’s participants (i.e., teenagers) makes comparison to the present data difficult. We contend that more rigorous investigation on the effects of HIFT on overall flexibility is warranted. 

Previous research has shown improvements in power which were not observed within the present study. While Sobero et al. [20] noted improvements in both upper (medicine ball put; 86.6 ± 12.8 vs. 94.6 ± 12.7 in) and lower body (vertical jump; 11.3 ± 3.4 vs. 14.2 ± 2.5 in) power following 10 weeks of HIFT, these data do not corroborate their findings with respect to upper body power. Interestingly, one potential reason for these discrepancies is perhaps the unit of measurement chosen (meters versus inches, respectively) for analysis. A smaller unit of measurement could yield smaller margins of error for statistical estimates. Regardless, the imprecision and insensitivity of using distance as the primary assessment of muscular power should be rectified by measuring velocity of a known resistance (force) during a given task (i.e., barbell bench press) in future studies. 

Muscular endurance improved across both sexes within the present study. However, females improved significantly for the push-up test while males improved for the pull-up test. While others [20,43] have noted improvement in upper body muscular endurance following HIFT, none have reported differences between sexes with respect to improvement in specific movements (i.e., pushing versus pulling). This finding could be due to a potential ceiling effect within the male participants for the push-up exercise. While these data support previous work for improvements on the push-up test [20], no studies could be identified that utilized the pull-up test as an outcome following a HIFT intervention. 

Muscular strength gains of the current study are similar to what McKenzie found in 2015, however the current results demonstrate increases in multiple 1RM scores for HIFT-trained men and women [43]. McKenzie found that inexperienced HIFT women (n = 10) improved 1RM back squat 23.1% after one month of CrossFit training [43]. After six weeks of HIFT training, Sobrero et al. reported improved muscular strength within the 1RM bench press test [20]. Within the present study only the 1RM back squat test is statistically different from baseline for female participants, while improvements across all tests of 1RM strength for all groups was observed. 

Though improvements in aerobic capacity were observed in the current study for less experienced female HIFT participants, there is little agreement on the effects of HIFT on cardiovascular fitness. For example, Crawford et al. reported no statistically significant improvements in untrained adults after nine weeks of HIFT training [23]. Contrastingly, Nieuwoudt and colleagues suggested improvements of exercise capacity (2.43 ± 0.12 vs. 2.81 ± 0.15 L/min) in sedentary adults with type 2 diabetes after a six-week CrossFit intervention [44]. Finally, Sobrero et al. found no significant change in cardiorespiratory fitness after six weeks of HIFT training in eight recreationally active women [20].

Physiologically, the disparate effects of varying HIFT experience levels on fitness variables warrant further consideration. The increase in muscular strength and aerobic capacity observed within the novice (0–6 months) experience group reflects a typical improvement time-course for these physiological variables. While the early mechanisms of muscular strength increase are thought to be predominantly neural with hypertrophy acting as the driver in later stages of training [45], contemporary hypotheses continue to highlight the impact of neural adaptations in later stages of training experience (e.g., 7+ months) [46]. These findings can explain why we see continued improvement in muscular strength outcomes across experience groups, yet smaller magnitudes. As could be expected [47], we observed increases in aerobic endurance (evidenced by 1.5 mile run time) within the 0–6-month group with relative decreases in the 7+ months group. Two potential mechanisms for this contrast are plausible. First, the greatest amount of change in aerobic capacity is noted within the first six months of training [47]. After this time increases in exercise volume are recommended to see further improvement. HIFT, by design, is a time-efficient training modality. Thus, one could posit that without increased volume of HIFT it would be unlikely to observe significant increases beyond the initial novice experience group. It is likely that adaptations to HIFT may not necessarily result in continual improvement across all fitness domains. Specifically, Crawford et al. [23] have recently demonstrated that changes in physical work capacity are not related to changes in the associated physiologic measures of fitness (i.e., change in aerobic capacity does not predict improvement in one’s ability to perform greater work as a result of HIFT interventions). In fact, within that study, aerobic capacity was negatively correlated with work capacity improvement [22]. Authors hypothesized that the primary outcome of HIFT may be greater synchronization and integration of physiologic systems rather than continual improvement. Such integration could potentially result in greater movement economy during tests used in the present study such as the 1.5-mile run test without meaningful increases in maximal aerobic capacity. 

Considering the large number of individuals that currently do not meet the physical activity guidelines, HIFT may be a viable option. It is encouraging to see significant increases in multiple fitness domains even with increasing HIFT training age, with no significant fitness declines. A growing body of research supports HIFT as a training modality that can be modified to any fitness level, and has been shown to improve cardiovascular endurance, strength, and flexibility [30,48], and our findings add muscular endurance and power to that list. Our findings are also important from a public health perspective, given that ‘lack of time’ continues to be one of the most frequently reported barriers to regular physical activity participation [49], and as a concurrent type of exercise combining aerobic and muscle strengthening activities the workouts are considerably shorter than those that separate those modalities [29]. Moreover, exercise participation to HIFT has been positively correlated to a number of factors, including enjoyment [29,50,51] intrinsic motivation [29,50], and social support [29,52].

Strengths of the current study include the variety of HIFT experience among participants and the measurement of multiple fitness domains. Due to the modifications needed for some participants, the numbers per assessment and category varied, likely affecting the power to identify significant differences. A primary limitation of the current study is the lack of randomization and control as well as blinding of researchers during measurements, as well as the potential variation in stimulus participants were exposed to during their HIFT workouts. However, since the primary purpose of this study was to evaluate the benefits of HIFT as a program, it was not integral to the research design to randomize. Participants’ attendance ranged from two to five days per week, and the authors did not control for factors like physical activity and changes in diet. Not all participants were able to complete all fitness tests due to pre-existing injuries or minor injuries sustained during the study period (both outside of and during HIFT training). Future research should follow more participants for a longer period of time using the information from this study to evaluate in a more detailed, controlled manner to elucidate the physiological mechanisms of change. 

## 5. Conclusions

The results of the current study indicate the positive effects of HIFT program participation on multiple fitness domains with greater improvements for women with less experience. Future research should elucidate the mechanisms responsible for these adaptations. 

## Figures and Tables

**Table 1:** Demographic and anthropometric characteristics of CrossFit program participants by length of experience (n = 45).

Characteristic	0–6 Months				7+ Months			
	Women (n = 11)		Men (n = 13)		Women (n = 12)		Men (n = 9)	
	Mean (SD)	N (%)	Mean (SD)	N (%)	Mean (SD)	N (%)	Mean (SD)	N (%)
Age (years)	30.2 (13.2)	--	26.5 (11.7)	--	37.3 (15.7)	--	33.3 (11.8)	--
Height (m)	1.65 (0.05)	--	1.82 (0.09)	--	1.77 (0.07)	--	1.78 (0.06)	--
Weight (kg)	65.7 (11.9)	--	81.4 (12.5)	--	59.8 (4.4)	--	79.9 (8.0)	--
Body Mass Index (kg/m^2^)	24.3 (4.7)	--	24.6 (2.6)	--	21.8 (1.3)	--	25.2 (3.1)	--
HIFT experience (months)	2.6 (2.6)	--	3.5 (2.1)	--	18.7 (5.9)	--	16.0 (5.0)	--
HIFT participation (days per week)	4.0 (1.2)	--	4.1 (1.3)	--	3.8 (0.9)	--	4.0 (1.0)	--
2	--	2 (18.2)	--	2 (15.4)	--	--	--	--
3	--	1 (9.1)	--	3 (23.1)	--	6 (50.0)	--	3 (33.3)
4	--	3 (27.3)	--	1 (7.7)	--	2 (16.7)	--	4 (44.4)
5	--	5 (45.5)	--	6 (46.2)	--	4 (33.3)	--	1 (11.1)
6			--	1 (7.7)			--	1 (11.1)

**Table 2:** Components of the three fitness measurement sessions linked to 10 physical skills.

Session	Fitness Measure	10 Physical Skills of General Physical Preparedness
1	Agility Hop Test	Balance, Coordination, Accuracy
	Pro-Agility Test	Agility
	Novel Flex-Tester^®^ Trunk Flexibility Tester (i.e., Sit and Reach)	Flexibility
	Standing Long Jump; Medicine Ball Put	Power
	Maximum # of repetitions in 60 s for each of the following: Push-ups, Sit-ups, Body Weight Squats Maximum # of Pull-ups	Stamina/Muscular Endurance
2	1 Repetition Maximum for each of the following: Back Squat, Standing Press, Deadlift	Strength
3	400 Meter Sprint	Speed Cardiovascular/Respiratory Endurance
	Cooper [31] 1.5 Mile Run/Walk Test	

Note: Some women completed push-ups on their knees. Some women and men completed modified pull-ups using an elastic band for assistance.

**Table 3:** Fitness changes from baseline to 6 months by experience groups for women.

Test	0–6 Months Experience (n = 11)		7+ Months Experience (n = 12)		Group × Time		
	Pre-test M (SD)	Post-test M (SD)	Pre-test M (SD)	Post-test M (SD)	F (dof)	*p*-Value	η^2^
Agility Hop Test (errors)	5.8 (2.5)	6.1 (2.5)	6.3 (3.0)	4.9 (1.8)	1.91 (1,21)	0.182	0.083
Pro-agility Test (seconds)	5.9 (0.8)	6.0 (0.9)	5.8 (0.5)	5.9 (0.6)	0.16 (1,21)	0.698	0.007
Sit-and-Reach (cm)	30.7 (10.4)	32.5 (9.9) **	31.0 (9.2)	34.7 (7.6) **	1.62 (1,21)	0.217	0.072
Standing Long Jump (m)	1.67 (0.38)	1.74 (0.37) *	1.80 (0.27)	1.85 (0.25) *	0.24 (1,21)	0.626	0.011
Seated Medicine Ball Put (m)	2.49 (0.32)	2.53 (0.32)	2.33 (0.30)	2.41 (0.27)	0.39 (1,21)	0.541	0.018
Push-ups in 60 sec (reps)	25.7 (5.5) ^4^	31.3 (3.4) *	25.0 (7.9) ^5^	28.1 (9.6) *	0.47 (1,12)	0.506	0.038
Knee Push-ups in 60 sec (reps)	23.2 (6.5) ^3^	28.6 (8.4)	31.3 (7.1) ^2^	29.8 (3.8)	3.89 (1,7)	0.089	0.357
Sit-ups in 60 sec (reps)	39.2 (13.0)	41.6 (14.7)	41.0 (7.2)	41.6 (6.7)	0.73 (1,21)	0.402	0.034
Squats in 60 sec (reps)	40.3 (10.8)	41.8 (11.5)	46.3 (5.3)	46.8 (5.6)	0.26 (1,21)	0.614	0.012
Pull-ups (reps)	3.5 (3.5) ^1^	5.0 (4.2)	6.5 (3.9) ^2^	7.8 (3.5)	0.04 (1,4)	0.859	0.009
Band-assisted Pull-ups (reps)	6.7 (6.0) ^6^	8.4 (5.9)	13.4 (6.7) ^5^	14.5 (6.6)	0.18 (1,15)	0.677	0.012
1 Rep Max Back Squat (kg)	56.2 (8.6) ^6^	64.7 (13.8) ***	56.4 (14.4)	60.3 (15.0) ***	3.37 (1,19)	0.082	0.150
1 Rep Max Press (kg)	30.0 (6.4)	32.7 (9.3)	31.8 (5.9)	33.1 (6.2)	1.37 (1,21)	0.255	0.061
1 Rep Max Deadlift (kg)	71.6 (19.4)	79.0 (27.9) **	80.8 (20.4)	86.6 (19.8) **	0.13 (1,21)	0.727	0.006
400 Meter Run (min)	1:29 (0:11) ^7^	1:31 (0:14)	1:28 (0:13)	1:30 (0:12)	0.15 (1,19)	0.707	0.008
1.5 Mile Run (min)	12:26 (1:13) ^7^	12:04 (1:21)	12:15 (1:19)	12:40 (1:33)	4.48 (1,9)	0.048	0.191
Estimated VO_2_max	42.0 (3.7) ^5^	42.3 (3.1)	43.3 (3.9)	44.0 (3.8)	0.09 (1,17)	0.771	0.005

Note: * *p* < 0.05, ** *p* < 0.01, *** *p* < 0.001, main effect for time; ^1^ n = 2. ^2^ n = 4. ^3^ n = 5. ^4^ n = 6. ^5^ n = 8. ^6^ n = 9. ^7^ n = 10. dof = degrees of freedom.

**Table 4:** Fitness changes from baseline to 6 months by experience groups for men.

Test	0–6 Months Experience (n = 13)		7+ Months Experience (n = 9)		Group × Time		
	Pre-test M (SD)	Post-test M (SD)	Pre-test M (SD)	Post-test M (SD)	F (dof)	*p*-Value	η^2^
Agility Hop Test (errors)	4.5 (3.2)	4.2 (2.7)	4.9 (2.7)	3.7 (1.9)	0.90 (1,20)	0.353	0.043
Pro-agility Test (seconds)	5.2 (0.4)	5.2 (0.4)	5.1 (0.4)	5.1 (0.4)	0.02 (1,20)	0.894	0.001
Sit-and-Reach (cm)	28.0 (8.0)	29.7 (7.5) *	30.5 (10.2)	34.0 (7.7) *	0.71 (1,20)	0.408	0.034
Standing Long Jump (m)	2.30 (0.28)	2.30 (0.27)	2.40 (0.22)	2.37 (0.18)	0.68 (1,20)	0.418	0.033
Seated Medicine Ball Put (m)	3.13 (0.42)	3.15 (0.32)	3.02 (0.36)	3.08 (0.43)	0.26 (1,20)	0.615	0.013
Push-ups in 60 sec (reps)	36.9 (11.8)	38.3 (11.8) *	39.1 (14.4)	38.9 (11.3) *	0.41 (1,20)	0.530	0.020
Sit-ups in 60 sec (reps)	45.2 (8.6)	45.4 (7.1)	46.8 (10.1)	49.7 (7.8)	1.16 (1,20)	0.295	0.055
Squats in 60 sec (reps)	49.8 (11.6)	50.5 (7.3)	53.3 (10.3)	53.9 (9.9)	0.01 (1,20)	0.946	0.000
Pull-ups (reps)	8.1 (3.1) ^4^	10.2 (5.0) *	11.6 (4.1) ^3^	13.0 (3.4) *	0.24 (1,15)	0.632	0.016
Band-assisted Pull-ups (reps)	6.2 (4.2) ^2^	7.6 (4.9)	2.0 (1.4) ^1^	4.5 (2.1)	0.27 (1,5)	0.624	0.052
1 Rep Max Back Squat (kg)	100.5 (25.8)	109.0 (28.6) ***	103.1 (30.4)	112.7 (33.7) ***	0.11 (1,20)	0.748	0.005
1 Rep Max Press (kg)	51.3 (10.0)	54.4 (11.5)	54.8 (12.5)	54.8 (13.7)	3.23 (1,20)	0.087	0.139
1 Rep Max Deadlift (kg)	123.6 (28.5)	130.4 (26.2) ***	139.7 (35.1)	150.8 (36.0) ***	0.92 (1,20)	0.348	0.044
400 Meter Run (min)	1:11 (0:11) ^5^	1:11 (0:07)	1:13 (0:14)	1:11(0:07)	0.52 (1,18)	0.479	0.028
1.5 Mile Run (min)	11:30 (1:41) ^5^	11:08 (2:00)	11:08 (2:18)	11:12 (2:27)	0.65 (1,18)	0.430	0.035
Estimated VO_2_max	45.1 (8.7) ^5^	46.4 (9.6)	48.2 (7.6)	48.5 (6.9)	0.71 (1,18)	0.412	0.038

Note: * *p* < 0.05, ** *p* < 0.01, *** *p* < 0.001, main effect for time; ^1^ n = 2. ^2^ n = 5. ^3^ n = 7. ^4^ n = 10. ^5^ n = 11. dof = degrees of freedom.
